# Autologous immune system reset for kidney allograft acceptance: mechanisms, evidence, and a testable reconstitution-window model

**DOI:** 10.3389/fmed.2026.1895856

**Published:** 2026-08-05

**Authors:** Chunhui Lyu, Zhishui Chen, Daqiang Zhao

**Affiliations:** 1 Institute of Organ Transplantation, Tongji Hospital, Tongji Medical College, Huazhong University of Science and Technology, Wuhan, China; 2 Key Laboratory of Organ Transplantation, Ministry of Education, Wuhan, China; 3 NHC Key Laboratory of Organ Transplantation, Wuhan, China; 4 Key Laboratory of Organ Transplantation, Chinese Academy of Medical Sciences, Wuhan, China; 5 Hubei Provincial Clinical Research Center for Organ Transplantation, Wuhan, China

**Keywords:** autologous hematopoietic stem cell transplantation, donor-specific hyporesponsiveness, immune acceptance, immune reconstitution-window, immune system reset, immunosuppression minimization, kidney transplantation, operational tolerance

## Abstract

Autologous hematopoietic stem cell transplantation (AHSCT)-based immune system reset (ISR) is conceptually distinct from donor-hematopoietic chimerism: recipient immunity is deeply depleted and rebuilt from recipient-derived hematopoietic stem/progenitor cells, without intended donor hematopoiesis. This review evaluates whether that platform could support kidney-allograft acceptance under reduced immunosuppression and, in exceptional responders, justify later testing of operational tolerance. We separate four evidence levels: mechanistic observations after AHSCT for autoimmune disease, preclinical hematopoietic-rescue models without intended donor chimerism, human partial-reset/minimization studies, and true drug-free tolerance. Autoimmune-disease data establish repertoire renewal and regulatory rebalancing, but do not prove alloantigen-specific tolerance. Experimental and early clinical transplant evidence remains limited and heterogeneous. We refine the proposed immune reconstitution-window model as a falsifiable framework in which continuous graft-derived donor-antigen exposure must coincide with a transient period of heightened immune plasticity. Donor antigen may enter this window through kidney transplantation during reconstitution after pretransplant ISR, or may already be present when ISR is applied to a stable recipient with an established allograft. Antigen presence is hypothesized to be necessary, but not sufficient, for donor-specific hyporesponsiveness. The model is linked to measurable targets: reduction of circulating and memory lymphocytes, turnover of donor-reactive clones, recovery of thymic output and naive repertoires, HLA-antibody trajectories interpreted against pretransplant donor-specific antibody status and specificity, absence of molecular or histologic graft injury, and reduced immunosuppression requirements. We also address resistant plasma cells, tissue-resident memory, pathogen-specific immunity, natural-killer-cell missing-self responses, age-dependent thymic recovery, stem-cell source, organ- and donor-type constraints, and kidney-specific safety. Current evidence supports biomarker-gated mechanistic testing in highly selected patients, not routine AHSCT or unmonitored immunosuppression withdrawal.

## 1 Introduction

Long-term kidney-allograft survival remains limited by chronic alloimmune injury and cumulative complications of maintenance immunosuppression. Operational tolerance–stable graft function without maintenance immunosuppression and without progressive alloimmune injury–has been observed, but remains rare and difficult to induce reproducibly (1, 2). International tolerance workshops therefore emphasize cautious endpoint selection, mechanistic monitoring, and predefined rescue rules (3).

Most clinical tolerance programs have used mixed or full donor-hematopoietic chimerism, in which donor-derived hematopoiesis can impose central and peripheral tolerance (4). Autologous hematopoietic stem cell transplantation (AHSCT)-based immune system reset (ISR) is biologically different. It reinfuses recipient-derived hematopoietic stem/progenitor cells (HSPCs), does not intend donor chimerism, and therefore seeks to modify the recipient immune repertoire rather than replace it with a donor-derived immune system. The absence of intended donor hematopoiesis reduces classical graft-vs.-host disease risk, but it also removes the strongest established mechanism of donor-specific central tolerance.

AHSCT in severe autoimmune disease demonstrates that pathogenic immune states can be modified by immune-cell depletion followed by structured reconstitution (5–7). The transplant question is narrower and more demanding: can a recipient-derived immune system, rebuilt while a vascularized kidney allograft supplies donor antigen, acquire measurably reduced donor reactivity without exposing the patient or graft to unacceptable conditioning, infection, or lymphopenic-rebound risk? This review distinguishes mechanistic plausibility from preclinical evidence, human partial-reset experience, and true tolerance evidence; it then proposes a testable, kidney-specific roadmap.

## 2 Definitions, clinical endpoints, and evidence hierarchy

For this review, operational tolerance denotes stable kidney-allograft function without maintenance immunosuppression and without biochemical, molecular, histologic, or serologic evidence of progressive alloimmune injury. Immune acceptance denotes stable graft function under materially reduced and strategically simplified immunosuppression, with controlled alloimmunity and acceptable safety. Acceptance is not equivalent to tolerance, but it is a clinically meaningful endpoint because it may reduce lifetime calcineurin-inhibitor, glucocorticoid, and antimetabolite exposure without requiring premature complete withdrawal (1–3).

Autologous ISR is a platform comprising immune debulking and recipient-derived immune rebuilding. Here, immune debulking is used only when its targets are specified: circulating T and B cells, donor-reactive memory clones, tissue-resident memory cells, plasmablast/plasma-cell compartments, and inflammatory antigen-presenting cells are neither equally accessible nor equally depleted. In this review, recipient immune re-education means a hypothesized post-reconstitution state in which responses to donor antigens are measurably reduced while third-party and pathogen responses are relatively preserved. It is not directly observable and must be inferred from reduced donor-reactive T-cell responses, turnover rather than preferential expansion of pre-existing alloreactive clones, HLA-antibody trajectories interpreted against pretransplant DSA status and specificity, stable graft-injury markers and histology, and a sustained reduction in immunosuppression requirement.

Four evidence categories are kept separate throughout: (i) human mechanistic evidence after AHSCT for autoimmune disease; (ii) preclinical hematopoietic-rescue models without intended donor chimerism, with the exact cell source specified; (iii) human partial-reset or immunosuppression-minimization studies in solid-organ transplantation; and (iv) true operational-tolerance studies. These categories are not interchangeable. In particular, remission of autoimmunity and prolonged allograft survival in rodents provide biological plausibility but do not establish donor-specific clinical tolerance in kidney recipients.

This hierarchy also determines what can reasonably be claimed from each endpoint. A fall in total lymphocyte count shows that depletion occurred, but does not establish that the intended cellular compartments were adequately removed or that immune reset occurred; recovery of naive cells and repertoire diversity is reconstitution, not donor specificity; a lower donor-reactive response than baseline is mechanistic evidence, not proof of graft protection; and stable creatinine alone cannot exclude subclinical antibody-mediated or molecular injury. Conversely, complete drug withdrawal is not the only informative endpoint. A sustained reduction in immunosuppressive exposure accompanied by concordant cellular, humoral, molecular, histologic, and functional stability would be a meaningful intermediate success. This graded framework is intended to prevent both overinterpretation of early mechanistic signals and underrecognition of clinically valuable minimization.

## 3 What autologous ISR is–and what it is not

A complete AHSCT-based ISR procedure includes candidate selection, HSPC mobilization and collection, conditioning with chemotherapy and/or serotherapy, autologous HSPC reinfusion, supportive care, antimicrobial prophylaxis, revaccination planning, and longitudinal immune surveillance (5, 8, 9). Partial ISR uses selective or combined depletion of T cells, B cells, and/or broader lymphocyte compartments without stem-cell rescue. It may be safer and more deployable, but tissue-resident memory T cells and long-lived plasma cells can persist, and homeostatic expansion can preferentially repopulate residual memory clones (6, 7).

Mobilized peripheral-blood stem cells (PBSCs) are the standard autologous source because collection is scalable and neutrophil and platelet recovery are generally faster than after marrow harvest (8–10). Bone marrow avoids pharmacologic mobilization and may contain fewer circulating mature lymphocytes, which is theoretically attractive if reinfusion of pathogenic memory is a concern; however, it requires an invasive harvest, commonly yields fewer CD34-positive cells, and can delay hematopoietic recovery (10). No direct study has compared PBSC vs. marrow as the optimal graft source for tolerance-oriented autologous ISR. Therefore, graft source should be treated as an experimental variable, with product phenotyping and, if used, *ex vivo* lymphocyte depletion reported explicitly.

Resetting alloimmunity also perturbs useful immunity. Conditioning and serotherapy can reduce pathogen-specific cellular and humoral memory; post-transplant revaccination is therefore recommended because protective immunity may be lost or substantially weakened after AHSCT (5, 8, 11). Kidney recipients additionally remain exposed to maintenance immunosuppression, latent cytomegalovirus (CMV), Epstein–Barr virus (EBV), BK polyomavirus, hepatitis viruses, and dialysis-related infection risk. These liabilities are part of the intervention rather than generic background toxicity and must be incorporated into candidate selection, prophylaxis, vaccination timing, immune-response testing, and stop rules.

Autologous ISR must also remain distinct from donor-chimerism strategies. CD117/c-Kit-directed approaches primarily deplete HSCs and open marrow niche space, with relatively limited direct effects on mature lymphocytes (12, 13). CD45-directed constructs are different because CD45 is expressed on HSPCs and most nucleated hematopoietic cells; depending on construct, dose, antigen density, and tissue penetration, they can combine HSC niche clearance with broader depletion of mature lymphoid and myeloid compartments (14, 15). Thus, a transplant-adapted autologous ISR regimen should be selected for its measured compartment-specific immune effects, not simply for donor-engraftment capacity.

Complete and partial ISR also differ in timing and recoverability. A complete AHSCT procedure creates a predictable hematologic nadir and requires stem-cell rescue, but it permits systematic sampling of the graft product, depletion depth, engraftment, and subsequent repertoire reconstruction. Partial ISR avoids high-dose conditioning and may be repeated or combined with standard induction, yet its biological boundary is less clear because residual cells can dominate recovery. Future studies should report the mobilization regimen, CD34-positive cell dose, graft manipulation, conditioning exposure, serotherapy pharmacokinetics, absolute subset nadirs, tissue-compartment surrogates, and time to neutrophil, platelet, and lymphocyte recovery (5, 8, 9). Without these parameters, negative and positive studies cannot be compared mechanistically.

## 4 Lessons from autoimmune disease: biological proof of reset, not proof of allo-tolerance

### 4.1 Repertoire renewal, thymic output, and regulatory recovery

The strongest human mechanistic evidence comes from multiple sclerosis (MS). After AHSCT, thymic output contributes newly generated clonotypes to a more diverse T-cell receptor (TCR) repertoire, while homeostatic expansion of surviving clones also contributes to early recovery (16, 17). B-cell receptor (BCR) studies in systemic sclerosis show a shift toward a more naive repertoire after myeloablative AHSCT (18). Favorable clinical courses have also been associated with regulatory-cell recovery and reduced inflammatory programs (19, 20). These observations demonstrate immune reconstruction, but not necessarily deletion of all pathogenic memory or stable antigen-specific tolerance.

The autoimmune analogy has a fundamental boundary. Autoantigens are self, persist throughout development, and are embedded in central-tolerance pathways. Alloantigens are genetically foreign and are recognized through direct, indirect, and semi-direct pathways of T-cell allorecognition (21). They are also commonly encountered during ischemia-reperfusion and surgical inflammation. The relevant transplant hypothesis is therefore not that AHSCT automatically creates tolerance, but that sufficient reduction of mature donor-reactive memory followed by thymic and peripheral repertoire renewal may produce measurable donor-specific hyporesponsiveness under controlled antigen exposure.

Mechanistic sampling must distinguish newly generated cells from phenotypic reversion or expansion of surviving cells. T-cell receptor excision circles (TRECs) and recent-thymic-emigrant phenotypes can support thymic output, but should be interpreted with age, cell proliferation, and absolute counts (16, 22). TCR/BCR sequencing should quantify richness, evenness, clonal persistence, and turnover (16–18), ideally linking donor-reactive cells identified by activation-induced markers, cytokine capture, peptide-HLA reagents, or mixed-lymphocyte assays to specific clonotypes. Regulatory-cell frequency alone is insufficient; suppressive function, stability, and the effector-to-regulatory balance are more informative (19, 20). Similarly, numerical B-cell recovery dominated by transitional or naive cells does not establish elimination of marrow or tissue plasma cells (18).

### 4.2 Clinical efficacy, toxicity, and the problem of relapse

Randomized and cohort studies show that AHSCT can provide durable disease control in selected aggressive autoimmune diseases, but benefit is neither universal nor cost-free. In relapsing MS, nonmyeloablative AHSCT improved disease control in the Multiple Sclerosis International Stem Cell Transplant (MIST) trial (23), while long-term cohorts support greatest benefit in inflammatory disease before irreversible tissue injury dominates (24, 25). In systemic sclerosis, the Autologous Stem Cell Transplantation International Scleroderma (ASTIS), Scleroderma: Cyclophosphamide or Transplantation (SCOT), and Autologous Stem-cell Systemic Sclerosis Immune Suppression Trial (ASSIST) studies demonstrated clinical benefit with different conditioning intensities (26–28), but also highlighted early treatment-related risk and the need for stringent cardiopulmonary selection (26, 27, 29). Crohn disease studies showed heterogeneous benefit and substantial toxicity (30–32). Systemic lupus erythematosus (SLE) series documented treatment-free remissions in selected patients but also relapse (33–35), whereas type 1 diabetes studies showed strong dependence on early intervention and residual beta-cell reserve (36–38).

Failure or relapse after AHSCT has direct relevance to kidney-allograft acceptance. Incomplete depletion of tissue-resident memory, survival of long-lived plasma cells, reinfusion of mature pathogenic cells with the autologous product, and homeostatic expansion of residual clones are recognized limitations of immune reset (6, 7). Limited thymic output, particularly in older patients, can further favor memory-dominated recovery (16, 22). In SLE, persistent humoral compartments and renewed inflammatory activation are plausible contributors to relapse (33–35). In kidney transplantation, analogous failure could appear early as lymphopenic-rebound rejection or later as dnDSA, chronic molecular injury, or recurrent alloreactive clonotypes. Thus, the trial question is not simply whether lymphocyte counts recover, but what cellular and clonal composition recovers.

The disease-specific trials also illustrate why a favorable average effect cannot be transferred directly to kidney transplantation. MS is predominantly a T-cell and B-cell repertoire disease in which neurologic inflammatory activity can be monitored clinically and radiographically; systemic sclerosis combines adaptive autoimmunity with vascular and fibrotic organ vulnerability; Crohn disease includes microbiome, epithelial, and stromal drivers that persist after reset; SLE frequently contains durable plasma-cell and immune-complex components; and type 1 diabetes requires sufficient target-tissue reserve. A kidney allograft combines foreign HLA, ischemic tissue, endothelial targets, maintenance immunosuppression, and latent infection. It therefore contains several failure modes simultaneously, arguing for multidimensional rather than single-compartment monitoring. Representative trials, toxicity signals, and their relevance to kidney-transplant ISR are summarized in Table 1.

**Table 1:** Representative AHSCT experience in immune-mediated diseases and its relevance to kidney-transplant ISR.

Clinical domain/study	Design and approximate sample	Conditioning intensity	Principal efficacy signal	Toxicity/limitation	Transferable lesson
Relapsing MS–MIST (23)	Randomized trial; *n* = 110	Nonmyeloablative cyclophosphamide plus ATG with autologous PBSC rescue	Lower risk of disease progression than continued disease-modifying therapy in selected active relapsing disease	Requires expert selection and prolonged infection/immune surveillance	Proof that immune debulking and reconstitution can alter a pathogenic adaptive repertoire; not allo-tolerance proof
Systemic sclerosis–ASTIS (26)	Randomized trial; *n* = 156	High-dose cyclophosphamide, rabbit ATG, CD34-selected autologous graft	Improved longer-term event-free outcomes vs. monthly cyclophosphamide	Early treatment-related deaths and major cardiopulmonary selection constraints	Reset depth must be balanced against nonhematologic toxicity in kidney candidates
Systemic sclerosis–SCOT (27)	Randomized trial; *n* = 75	Myeloablative cyclophosphamide, fractionated TBI, ATG, CD34-selected graft	Durable global-rank benefit in severe disease	Intensive regimen, irradiation toxicity, restricted eligibility	Biologically potent but difficult to justify in routine kidney recipients
Systemic sclerosis–ASSIST (28)	Open-label randomized phase 2; *n* = 19	Nonmyeloablative cyclophosphamide plus ATG	Improved skin and lung measures in a small selected cohort	Small study and limited generalizability	Lower-intensity platforms can retain biological activity but require confirmatory monitoring
Crohn disease–ASTIC/ASTIClite (30, 31)	Randomized trials; ASTIC, *n* = 45; ASTIClite, *n* = 23	ASTIC: intensive mobilization/conditioning; ASTIClite: lower-dose mobilization and reduced-intensity conditioning	Heterogeneous clinical benefit; reduced-intensity strategy tested to improve risk-benefit	Infection and regimen toxicity; nonimmune tissue drivers persist	Conditioning depth alone does not guarantee durable disease control
SLE registry and series (33–35)	Multicenter registry/series; tens of highly selected patients	Predominantly nonmyeloablative autologous regimens	Some durable treatment-free remissions	Relapse, infection, and selection bias	Residual humoral and memory compartments are plausible causes of failure
New-onset type 1 diabetes (36–38)	Early-phase and multicenter cohorts; *n* = 15–65	Nonmyeloablative cyclophosphamide plus ATG	Transient or durable insulin independence in a subset treated early	Benefit depends on residual beta-cell reserve; relapse occurs	Timing and target-organ reserve influence the value of immune reset

AHSCT, autologous hematopoietic stem cell transplantation; ASSIST, Autologous Stem-cell Systemic Sclerosis Immune Suppression Trial; ASTIC, Autologous Stem Cell Transplantation International Crohn Disease trial; ASTIClite, reduced-intensity ASTIC trial; ASTIS, Autologous Stem Cell Transplantation International Scleroderma trial; ATG, anti-thymocyte globulin; MIST, Multiple Sclerosis International Stem Cell Transplant trial; MS, multiple sclerosis; PBSC, peripheral-blood stem cell; SCOT, Scleroderma: Cyclophosphamide or Transplantation trial; SLE, systemic lupus erythematosus; TBI, total-body irradiation.

## 5 Conditioning for transplant-adapted ISR

### 5.1 Conditioning intensity and resistant compartments

Conditioning determines both reset depth and toxicity. Cyclophosphamide/anti-thymocyte globulin (ATG)-based lymphoablation aims to reduce mature adaptive immunity while preserving marrow function (8, 9). BEAM (carmustine, etoposide, cytarabine, and melphalan) plus ATG provides broader cytoreduction but increases mucositis, infection, and resource burden (8, 39). Irradiation-based myeloablation produces deeper repertoire disruption but has a narrow safety margin in patients with chronic kidney disease and cardiovascular comorbidity (27, 29).

No clinically acceptable regimen should be assumed to eradicate all immune memory. Tissue-resident memory T cells, long-lived plasma cells, and inflammatory myeloid programs may survive or recover rapidly (6, 7). Lymphopenia can also drive homeostatic expansion of residual donor-reactive clones (7, 39). Reset depth must therefore be measured using absolute counts, phenotype, clonotype turnover, donor-reactive functional assays, antibody kinetics, and graft-injury readouts rather than inferred from total lymphocyte depletion alone (6, 40).

Kidney disease alters conditioning risk and drug handling. Reduced renal clearance, dialysis timing, fluid balance, endothelial dysfunction, and pre-existing cardiopulmonary disease can change exposure to cyclophosphamide metabolites, antimicrobial agents, and supportive drugs. The regimen used successfully in a young patient with inflammatory MS cannot be imported unchanged into an older dialysis population. A kidney-ISR dose-escalation study should therefore begin with pharmacokinetic and organ-toxicity objectives, use renal-adjusted supportive protocols, and separate the minimal exposure required for measurable donor-reactive memory reduction from the maximal exposure tolerated. Conditioning intensity should be escalated only after lower-intensity cohorts fail predefined biological endpoints, not merely because complete withdrawal was not achieved.

### 5.2 Immune debulking vs. HSC niche clearance

For donor-chimerism protocols, HSC niche clearance creates space for donor HSPC engraftment. For autologous ISR, the primary tolerance-oriented objective is reduction of mature pathogenic/alloreactive compartments, although autologous HSPC rescue may still be required after intensive conditioning. CD45 is expressed on HSPCs and most mature leukocytes. Accordingly, CD45-directed immunotoxins or antibody-drug conjugates can combine HSC depletion/niche opening with broad immune debulking, including mature lymphoid and myeloid cells, as shown in preclinical models (14, 15). The actual depth in T cells, B cells, NK cells, monocytes/macrophages, granulocytes, and plasma-cell subsets will depend on target density, construct pharmacology, dose, and tissue access. Bone-marrow plasma cells are heterogeneous for CD45 expression, which generally decreases with maturation; CD45 targeting therefore cannot be assumed to eliminate all long-lived plasma cells (41). By contrast, c-Kit/CD117 strategies preferentially target HSCs and facilitate niche access, with much less direct effect on mature immune cells (12, 13).

A rational platform may require complementary modules, but the required combination depends on the depletion footprint of the base regimen. Rituximab primarily removes CD20-positive B cells and does not directly eliminate mature plasma cells, whereas ATG predominantly depletes T cells and can variably affect other lymphocyte subsets; in RESTARRT, the combination was used explicitly as a T- and B-cell-depletion strategy (42). Irradiation-based or cytotoxic regimens are less lineage-restricted, but depletion depends on dose, fractionation, shielding, and tissue compartment. Even marrow-lethal irradiation that requires hematopoietic rescue can leave radioresistant host T cells, so “lethal irradiation” does not imply complete immunologic ablation (43). Each module should therefore have a prespecified pharmacodynamic endpoint: T-cell and tissue-memory reduction, B-cell depletion, plasma-cell reduction when antibody production is targeted, myeloid/NK depletion when relevant, and HSPC rescue when conditioning requires it. Broad claims should be restricted to the compartments, tissues, and species in which depletion has actually been demonstrated.

### 5.3 Thymic integrity, age, and immune competence

The reconstitution-window hypothesis depends on thymopoiesis, yet conditioning can injure the same thymic substrate needed for repertoire renewal. Cyclophosphamide and ATG mainly reduce proliferating and circulating lymphocytes but can still alter thymic output through systemic inflammation and precursor depletion; total-body irradiation can damage thymic epithelial cells and stromal niches more directly. In AHSCT cohorts, recovery of T-cell receptor excision circles (TRECs), naive CD4-positive and CD8-positive T cells, and repertoire diversity differs by conditioning platform (7, 22). These measures should be incorporated into dose-finding rather than treated as exploratory endpoints.

Age is a central effect modifier because thymic involution reduces *de novo* naive T-cell output. Older kidney recipients may rely more heavily on peripheral expansion of surviving clones, increasing the risk that alloreactive memory dominates recovery. Initial trials should therefore restrict age and frailty, report biological rather than chronological immune age when possible, and prespecify failure of TREC/naive-cell recovery as mechanistic nonresponse. A regimen that achieves profound lymphopenia but prevents thymic recovery would contradict, rather than test successfully, the proposed model. The distinct effects of major conditioning platforms on immune debulking, niche clearance, and thymic recovery are summarized in Table 2.

**Table 2:** Conditioning platforms: distinction between immune debulking and HSC niche clearance.

Platform	Primary cellular objective	Immune-debulking effect	HSC niche-clearance effect	Thymic/immune-competence concern	Interpretation for kidney ISR
Cyclophosphamide plus ATG; autologous PBSC rescue (23, 26, 28)	Reduce proliferating and mature adaptive cells	Substantial circulating T-cell reduction; incomplete tissue memory and plasma-cell depletion	Variable; sufficient for autologous rescue but not designed for donor engraftment	Delayed naive T-cell recovery, infection, viral reactivation	Most plausible starting platform, but only with compartment-level pharmacodynamics and protective immunosuppression
BEAM plus ATG or comparable intermediate-intensity regimen (8, 39, 75)	Broader cytoreduction of memory compartments	Deeper adaptive-cell depletion than nonmyeloablative approaches	Moderate marrow ablation	Mucositis, infection, prolonged immune deficiency; potential thymic injury	Could test biological depth in exceptional candidates; high barrier to kidney-specific risk-benefit
Cyclophosphamide plus fractionated TBI plus ATG (27, 43)	Maximal repertoire disruption	Broad, dose-dependent multi-lineage cytoreduction; even marrow-lethal exposure may leave radioresistant host cells	High niche clearance	Direct thymic epithelial and organ toxicity	Unfavorable for routine kidney ISR; not an appropriate default clinical platform
CD45-directed antibody, immunotoxin, or antibody-drug conjugate (14, 15, 41)	Target HSPCs and mature CD45-positive leukocytes	Potentially broad depletion of T, B, NK, and myeloid compartments; plasma-cell effect varies with CD45 expression, maturation state, and tissue penetration	Substantial HSPC depletion and niche opening	Broad on-target immune deficiency; tissue access and lineage recovery must be measured	Dual-purpose niche-clearance and immune-debulking module, not an HSC-only strategy; currently preclinical for kidney ISR
CD117/c-Kit-directed depletion (12, 13)	HSC depletion/niche opening	Limited direct effect on mature lymphocytes, NK/myeloid cells, and plasma cells	Strong target-specific niche clearance	Progenitor depletion and uncertain effect on thymic precursor supply	Useful for engraftment biology, but insufficient as a stand-alone alloimmune reset
B-cell or plasma-cell-directed add-on (53)	Reduce antibody-producing lineage	B-cell depletion does not reliably eliminate long-lived plasma cells; plasma-cell strategies have distinct toxicity	Minimal	Hypogammaglobulinemia and infection	Should be selected according to whether the goal is rapid antibody reduction, suppression of ongoing production, or prevention of new antibody responses

ADC, antibody-drug conjugate; ATG, anti-thymocyte globulin; BEAM, carmustine/BCNU, etoposide, cytarabine, and melphalan; HSC, hematopoietic stem cell; HSPC, hematopoietic stem/progenitor cell; ISR, immune system reset; NK, natural killer; PBSC, peripheral-blood stem cell; TBI, total-body irradiation.

## 6 Evidence that hematopoietic reset without intended donor chimerism can modulate alloimmunity

### 6.1 Preclinical evidence without intended donor chimerism

Terminology is important in interpreting the experimental literature. Autologous transplantation means that cells are collected from and reinfused into the same individual. Syngeneic transplantation uses cells from a genetically identical individual, typically an animal of the same inbred strain. Congenic transplantation uses a donor and recipient with an otherwise closely matched genetic background but a defined genetic difference. “Host-type” is the wording used in the earliest rat study and indicates hematopoietic cells of the recipient's genetic type; it does not by itself establish that the cells were collected from that same animal (44–46). Accordingly, “recipient-derived” is reserved here for true autologous cells, whereas host-type, syngeneic, and congenic are retained only for the specific preclinical models that used those designs.

In the earliest rat cardiac-allograft study, lethally irradiated recipients repopulated with host-type hemopoietic cells achieved long-term graft survival (44). Subsequent rat studies explicitly used syngeneic hemopoietic cells or syngeneic bone marrow and showed that acceptance varied with class I and class II disparities (45, 46). In a later sensitized-rat model, syngeneic hematopoietic stem cell transplantation performed 2 months before kidney transplantation, combined with peri-transplant fludarabine and brief calcineurin inhibition, prolonged graft survival and yielded a subset of long-term survivors (47). Vascularized composite-allograft, autologous-marrow, and congenic hematopoietic stem cell transplantation studies provide additional evidence that hematopoietic rescue without intended donor chimerism can alter antibody-mediated injury or allograft survival (48–50). These models establish experimental tractability, but differ substantially in irradiation, timing, species, sensitization, adjunctive immunosuppression, and proof of donor specificity.

### 6.2 Human partial-reset experience and ongoing translation

The Immune Tolerance Network ITN039ST/RESTARRT trial is best classified as a human partial-reset and withdrawal study, not a general proof of tolerance. Ten living-donor kidney recipients received four doses of rabbit ATG and two doses of rituximab. Initial maintenance therapy comprised tacrolimus and sirolimus, with mycophenolate mofetil discontinued on day 12. In eligible participants, tacrolimus withdrawal was initiated between weeks 26 and 38 and completed over 4–8 weeks; after at least 26 weeks of sirolimus monotherapy, sirolimus withdrawal was initiated between weeks 56 and 88 and completed over 12–26 weeks. Six recipients completed withdrawal; four restarted therapy because of rejection or recurrent disease, one remained immunosuppression-free for more than 9 years, and one was lost to follow-up after 42 weeks off treatment (42). B cells recovered predominantly with a naive phenotype, whereas T-cell recovery remained memory-heavy. The limited durable success and divergent T- and B-cell recovery indicate that ATG-plus-rituximab-mediated T- and B-cell depletion was insufficient to ensure tolerogenic immune reconstruction in most recipients.

A pilot AHSCT study in liver recipients with recurrent primary sclerosing cholangitis used CD34-selected autologous cells after immunoablation with busulfan, cyclophosphamide, and rabbit ATG. Of five treated recipients, two achieved operational tolerance, but severe regimen-related morbidity and mortality made that specific protocol unsuitable for clinical adoption (51). The ongoing Immunological Reset to Allow Access to HLA-Compatible Transplantation (RESET) trial (ClinicalTrials.gov, identifier NCT06809075; EU CTIS 2023-506478-11-01) enrolls highly sensitized kidney candidates on dialysis. Its protocol includes apheresis with CD34-positive cell selection, nonmyeloablative conditioning using cyclophosphamide, rabbit ATG, and rituximab, followed by autologous CD34-positive HSPC rescue, with the objective of reducing HLA-antibody barriers and improving access to a compatible deceased-donor offer (52). Because outcome data are not yet available, RESET remains hypothesis-generating and should not be cited as efficacy evidence.

### 6.3 Humoral and innate barriers

An ISR regimen is not an immediate antibody-removal procedure and does not directly clear circulating DSA or non-HLA antibodies already present when the intervention begins. When rapid antibody reduction is required to achieve an acceptable crossmatch or control antibody-mediated injury, plasma exchange or immunoadsorption, with additional B-cell- or plasma-cell-directed therapy as appropriate, remains necessary (53). The more plausible humoral contribution of ISR is suppression of ongoing production by depleting memory B cells, plasmablasts, and, only when the conditioning agent reaches and effectively targets them, plasma cells. Even if antibody production is substantially suppressed, passive immunoglobulin decay is too slow and unpredictable to replace active antibody removal when an immediate reduction is clinically required; long-lived plasma cells and protected marrow or tissue niches can also sustain production. Antibody removal, suppression of continued production, and prevention of new antibody responses are therefore distinct therapeutic objectives.

The effect of ISR on innate immunity is regimen-dependent. Rituximab plus ATG mainly targets CD20-positive B cells and T cells, with variable effects on NK-cell subsets; it is not a broad myeloid-depletion platform. CD45-directed immunotoxins or antibody-drug conjugates can potentially reduce HSPCs and multiple mature leukocyte lineages, including NK and myeloid cells, if target expression and tissue penetration are sufficient (14, 15, 40). Irradiation-based or cytotoxic regimens can produce broader, dose-dependent depletion of innate and adaptive compartments, but even marrow-lethal exposure does not guarantee elimination of every tissue-resident or radioresistant population (43). Importantly, autologous reconstitution does not change donor-recipient missing-self genetics. Reconstituted recipient NK cells can therefore again respond when donor HLA class I fails to engage recipient inhibitory receptors, contributing to microvascular inflammation and synergizing with non-complement-fixing DSA in chronic antibody-mediated rejection (54, 55). NK-cell reconstitution, killer-cell immunoglobulin-like receptor/HLA relationships, endothelial injury, and microvascular-inflammation signatures should therefore be measured rather than assuming that adaptive reset captures all alloimmunity.

Solid-phase HLA-antibody assays can establish whether a DSA specificity was present in pretransplant serum and can follow the post-transplant trajectory of that specificity, but they cannot distinguish residual pretransplant immunoglobulin molecules from newly secreted antibody molecules of identical specificity. Trial endpoints should therefore be anchored to baseline DSA status. In recipients without DSA at baseline, absence of newly detected DSA is a clear humoral endpoint. For mechanistic clarity, initial post-transplant minimization studies should preferentially enroll such recipients. If recipients with baseline DSA are included, the protocol should prespecify acceptable decline or stability vs. a clinically meaningful rebound of the same specificity, account for assay variability, and interpret antibody kinetics together with complement-binding characteristics, microvascular inflammation, molecular findings, and antibody-mediated rejection. The same logic applies to non-HLA antibodies. Rapid reduction of clinically important baseline antibody still requires active removal; ISR should not be presented as a substitute. Plasma-cell-directed add-ons should be selected according to the intended objective and should not be applied automatically, because they increase infection risk and can obscure whether the base ISR regimen produced the observed biology.

## 7 A falsifiable immune reconstitution-window model

### 7.1 Biological rationale

**Figure 1:**
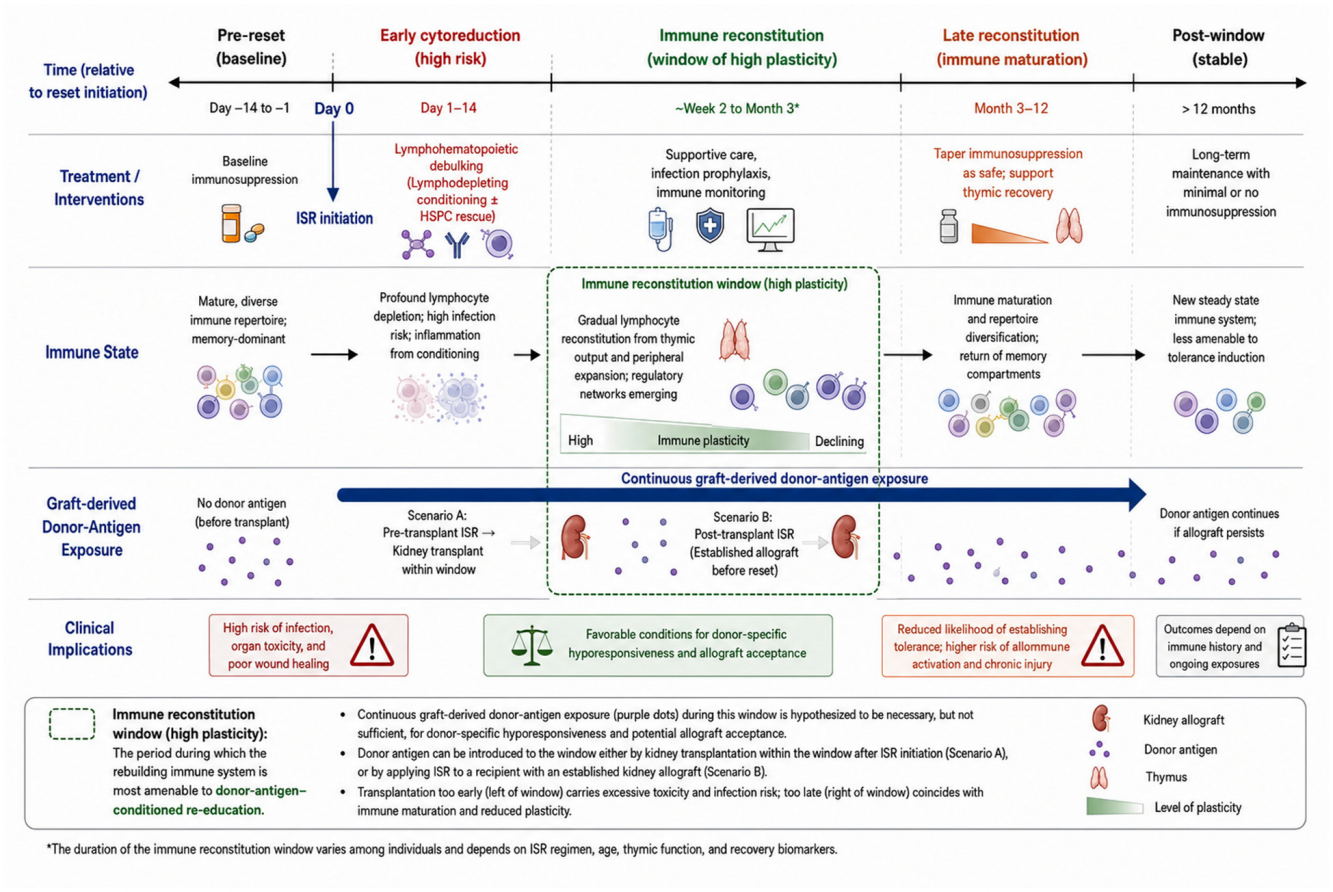
Testable immune reconstitution-window model for kidney-allograft acceptance after autologous immune system reset. The window denotes a transient phase of heightened immune plasticity during rebuilding, not a mandatory single surgical time point. Continuous graft-derived donor-antigen exposure during this interval is hypothesized to be necessary, but not sufficient, to bias reconstitution toward donor-specific hyporesponsiveness and potential allograft acceptance. Donor antigen may enter the window through either of two scenarios: (A) pretransplant ISR followed by kidney transplantation during reconstitution, once acute conditioning toxicity has begun to resolve but before immune maturation is largely complete; or (B) post-transplant ISR in a stable recipient with an established kidney allograft already present. The first pathway requires balancing perioperative safety against declining immune plasticity; the second avoids organ-offer timing but must overcome established donor-reactive memory, possible DSA, tissue-resident immunity, and pre-existing graft injury. The duration and quality of the window are expected to vary with ISR regimen, age, thymic function, inflammatory context, and recovery biomarkers. Antigen exposure alone is not tolerogenic: infection, graft injury, residual memory, or inadequate regulatory recovery may instead promote alloimmune activation. HSPC, hematopoietic stem/progenitor cell; ISR, immune system reset; DSA, donor-specific antibody; TREC, T-cell receptor excision circle.

We propose a finite period after autologous ISR during which the rebuilding recipient immune system is unusually plastic. Early recovery includes lymphopenia-driven expansion of residual cells, followed variably by naive T- and B-cell generation, thymic output, repertoire diversification, and regulatory recovery. The defining condition is not that kidney transplantation must occur at one fixed time after reset, but that graft-derived donor antigen is continuously present during this high-plasticity interval. This can be tested in two complementary scenarios: pretransplant ISR followed by kidney transplantation during immune reconstitution, after acute conditioning toxicity has subsided but before immune maturation is largely complete; or post-transplant ISR in a stable recipient whose established kidney allograft remains present throughout immune debulking and rebuilding. In either scenario, donor-antigen exposure is hypothesized to be necessary, but not sufficient, to bias the new repertoire toward donor-specific hyporesponsiveness. The temporal relationships are illustrated in Figure 1.

A vascularized allograft can provide antigen beyond the graft itself. Donor passenger leukocytes can migrate from the graft to recipient lymphoid organs (56). Recipient antigen-presenting cells can acquire intact donor HLA through cross-dressing and extracellular-vesicle transfer, supporting semi-direct presentation (57, 58). In experimental systems, circulating or peripheral dendritic cells can also home to the thymus and transport peripheral antigen (59–61). These findings make extra-graft and potentially thymic antigen availability biologically plausible. They do not prove that kidney-donor antigen reaches the adult human thymus in the quantity, form, and inflammatory context needed to delete donor-reactive thymocytes or induce durable regulation.

The inflammatory context may be as important as antigen presence. Ischemia-reperfusion injury, infection, delayed graft function, surgical complications, and rejection increase costimulation and inflammatory cytokines, potentially converting the same donor antigen from a tolerogenic to an immunogenic signal. For a pretransplant-ISR strategy, kidney transplantation at the deepest phase of cytopenia may be unsafe, whereas transplantation only after immune maturation is largely complete may reduce the opportunity for donor-antigen-conditioned re-education. A post-transplant-ISR strategy avoids the need to synchronize organ implantation with the window, because the established allograft supplies antigen throughout debulking and reconstitution; however, it must overcome pre-existing donor-reactive memory, possible DSA, tissue-resident immunity, and any chronic graft injury accumulated before reset. Protective immunosuppression during either pathway is therefore not conceptually opposed to reset: it may be required to restrain residual memory and inflammatory injury until favorable reconstitution is demonstrated. Minimization should begin only after objective evidence of graft stability and donor-specific hyporesponsiveness, and the model does not predict that immunosuppression should be withdrawn simply to “allow” tolerance.

### 7.2 Measurable predictions and falsification

The model predicts more than lymphocyte recovery. A successful biological window should show: (i) substantial reduction of pre-existing donor-reactive memory and high-frequency clonotypes; (ii) recovery of TRECs and naive T cells rather than only memory-dominated homeostatic expansion; (iii) BCR/TCR repertoire diversification without re-expansion of dominant donor-reactive clones; (iv) lower donor-reactive interferon-gamma enzyme-linked immunospot or proliferation responses relative to third-party responses; (v) no newly detected DSA in recipients without baseline DSA and no clinically meaningful rebound of tracked baseline DSA specificities with antibody-mediated graft injury in recipients who were DSA-positive before transplantation; and (vi) no contemporaneous molecular, histologic, or cell-free-DNA evidence of graft injury during immunosuppression reduction.

The model would be falsified, or the specific regimen judged biologically unsuccessful, if immune recovery remains memory-dominated, thymic output fails, donor-reactive clones rebound, DSA rises, NK/microvascular injury emerges, or graft injury precedes any safe reduction in immunosuppression. Demonstrating global immunodeficiency without donor-specific hyporesponsiveness would not constitute successful re-education.

## 8 Translational development in kidney transplantation

### 8.1 Candidate selection and comparison with lower-risk alternatives

AHSCT-based ISR should not be evaluated in a therapeutic vacuum. For sensitized candidates, kidney-paired donation, acceptable-mismatch or priority-allocation programs, conventional or plasma-cell-directed desensitization, and combinations of these approaches may achieve transplantation without the toxicity of stem-cell rescue (53, 62). For maintenance minimization, belatacept or other costimulation-blockade platforms can reduce calcineurin-inhibitor exposure, although rejection risk and patient selection remain important (63). ISR becomes ethically plausible only when its incremental mechanistic objective and anticipated benefit exceed these alternatives for a clearly defined population.

Initial kidney studies should favor patients with an otherwise poor risk-benefit balance but adequate physiological reserve: for example, highly sensitized dialysis candidates with prolonged waiting time who have exhausted allocation and desensitization options, or stable living-donor recipients enrolled in a mechanistic minimization study. Exclusion should include active infection, uncontrolled cardiovascular disease, limited pulmonary reserve, active malignancy, severe frailty, inadequate social support, or inability to comply with intensive surveillance.

### 8.2 Organ-specific and donor-type considerations

Kidney transplantation has dialysis as a rescue modality, but remaining on dialysis carries substantial mortality and morbidity, and dialysis-related comorbidity may also increase conditioning risk (64). Two kidney-specific translational pathways should be distinguished. In a pretransplant-ISR pathway, a living-donor setting is the most controllable initial design because vaccination, infection screening, HSPC collection, conditioning, donor sampling, and kidney transplantation can be coordinated so that graft-derived antigen enters the high-plasticity reconstitution window after acute toxicity has begun to resolve. A deceased-donor offer is harder to synchronize with that interval; RESET illustrates a wait-list design in which ISR is performed before a later compatible offer, but organ-offer uncertainty may expose a candidate to conditioning without transplantation (52). In a post-transplant-ISR pathway, a stable kidney allograft is already present and supplies donor antigen throughout immune debulking and reconstitution, avoiding organ-offer timing but introducing different barriers, including established donor-reactive memory, baseline DSA, chronic histologic injury, and the safety of conditioning an otherwise stable recipient. RESTARRT provides partial-reset experience in this post-transplant setting, although it did not use AHSCT (42). Both pathways remain investigational and should be evaluated separately rather than assumed to be biologically equivalent.

The liver is more amenable than the kidney to successful immunosuppression withdrawal in carefully selected stable recipients (65), but AHSCT for recurrent primary sclerosing cholangitis addresses recurrent immune-mediated disease as well as alloimmunity and used a highly toxic regimen (51). Heart and lung grafts provide less margin for a trial-related rejection episode: cardiac antibody-mediated rejection may present with allograft dysfunction and hemodynamic compromise, whereas acute lung rejection is a recognized risk factor for subsequent chronic lung allograft dysfunction (66, 67). Superimposing the infectious, cytopenic, and nonhematologic toxicity of conditioning further weakens the risk-benefit case for testing ISR first in these organs. Organ-specific reserve, consequences of rescue failure, and ability to biopsy repeatedly must therefore shape trial order; evidence should not be generalized across vascularized organs without qualification.

### 8.3 HLA-centered immunogenetic and complementary alloimmune risk stratification

HLA molecular mismatch and eplet analyses can enrich mechanistic stratification because higher class II molecular mismatch is associated with primary alloimmunity and dnDSA risk (68), while these metrics are also being investigated in tolerance-induction cohorts and clinical implementation frameworks (69, 70). However, simple eplet counts do not adequately weight differential eplet immunogenicity, expression, verification status, or recipient context; not all mismatches are biologically equivalent (71). Killer-cell immunoglobulin-like receptor/HLA combinations are an additional immunogenetic dimension relevant to NK-cell missing-self responses, but their prospective predictive value for ISR has not been established (54, 55). Pretransplant DSA specificity and strength, non-HLA antibodies, donor-reactive cellular assays, and molecular graft-injury measures are complementary alloimmune-risk variables rather than immunogenetic markers and should be analyzed alongside HLA-centered stratification.

### 8.4 Longitudinal monitoring and decision rules

Monitoring must reflect distinct biological phases. Before conditioning, trials should establish DSA and non-HLA antibody status, donor-reactive T-cell responses, TCR/BCR repertoire, lymphocyte subsets, immunoglobulins, TREC or equivalent thymic-output measures, vaccine serology, latent-virus status, kidney histology when available, estimated glomerular filtration rate (eGFR), proteinuria, and a molecular-injury baseline. During conditioning and engraftment, the priorities are hematologic recovery, infection/viral surveillance, immune-cell depletion pharmacodynamics, and avoidance of graft-directed tapering.

In the first month after kidney transplantation, dd-cfDNA can reflect ischemia-reperfusion injury and recovery rather than rejection alone; autologous ISR does not remove this confounding and may add conditioning-related inflammation (72). Early dd-cfDNA should therefore be interpreted as an individualized kinetic trajectory alongside creatinine, urine findings, Doppler imaging, infection tests, and clinical context, not as a stand-alone taper trigger. From approximately months 1–3, serial trends can be integrated with DSA, donor-reactive assays, and immune-reconstitution phenotyping. Molecular Microscope Diagnostic System or validated biopsy transcriptomics can add specificity when histology and dd-cfDNA are discordant, because dd-cfDNA is a marker of graft injury and correlates with both rejection and nonrejection injury states (73, 74).

The proposed longitudinal schedule and illustrative decision rules are summarized in Table 3. They are deliberately conservative and require prospective validation. Mechanistic success short of withdrawal can be defined as a durable reduction in donor-reactive cellular responses, demonstrable repertoire turnover with naive-cell/TREC recovery, no newly detected DSA in baseline-DSA-negative participants, no clinically meaningful rebound of tracked baseline DSA specificities with antibody-mediated graft injury in baseline-DSA-positive participants, stable protocol histology and molecular assessment, stable graft function, and a prespecified reduction in cumulative immunosuppression. Complete withdrawal is not required to demonstrate that the biological hypothesis has merit.

**Table 3:** Illustrative monitoring schedule and biomarker-gated action categories for a kidney ISR trial.

Phase/approximate timing	Core measurements	Findings supporting progression	Hold/reverse findings	Example action
Baseline before conditioning	DSA/non-HLA antibodies; donor-reactive ELISPOT/proliferation; TCR/BCR repertoire; subsets; immunoglobulins; TREC; vaccine serology; CMV/EBV/BK/hepatitis; eGFR/proteinuria; histology/molecular baseline when available	No active infection; acceptable organ reserve; measurable immune endpoints; protocol-defined immunologic indication	Active infection, uncontrolled comorbidity, irreversible graft injury, absent follow-up capacity	Do not proceed until reversible risks are corrected
Conditioning to engraftment (approximately day −10 to +30 relative to HSPC infusion)	Blood counts; lymphocyte/plasma-cell pharmacodynamics; fever/infection; CMV/EBV/BK; immunoglobulins; organ toxicity	Expected cell depletion and timely neutrophil/platelet recovery without invasive infection	Delayed engraftment, organ toxicity, uncontrolled viral replication, severe infection	No immunosuppression taper; intensify supportive care or terminate protocol intervention
Early kidney post-transplant period (0–1 month)	eGFR/creatinine trajectory; proteinuria; Doppler; DSA; infection; dd-cfDNA kinetics interpreted against ischemia-reperfusion injury; for-cause biopsy	Improving graft function; falling individualized dd-cfDNA trajectory; no newly detected DSA in baseline-DSA-negative recipients; no clinically meaningful rebound of tracked baseline DSA with graft injury; no rejection	Sustained dysfunction, newly detected DSA or clinically meaningful rebound of tracked baseline DSA, unexpected dd-cfDNA rise, clinical or histologic rejection	Biopsy when indicated; treat injury/rejection; maintain or restore protective immunosuppression
Early reconstitution (1–3 months)	T-cell/B-cell/NK subsets; naive-memory ratios; TREC; TCR/BCR turnover; donor-reactive vs. third-party responses; DSA; viral surveillance; dd-cfDNA trend	Naive/TREC recovery, repertoire renewal, reduced donor-reactive response, stable graft markers	Memory-dominant rebound, donor-reactive clonal expansion, newly detected or clinically meaningful rebound DSA, viral disease, or molecular injury	Continue full protection; classify as mechanistic nonresponse if persistent
Candidate minimization window (3–6 months or later; biomarker-defined)	All above plus protocol biopsy and molecular assessment; adherence and pharmacokinetics	Concordant cellular, humoral, molecular, histologic, and functional stability	Newly detected DSA or clinically meaningful rebound of tracked baseline DSA with graft injury, molecular/histologic rejection, sustained dd-cfDNA rise, or infection indicating inadequate immune competence	Only protocolized stepwise reduction; pause at first discordant signal
Long-term follow-up (6–12 months and annually)	DSA; function/proteinuria; dd-cfDNA trends; protocol/for-cause biopsy; molecular diagnostics; repertoire persistence; vaccine responses; malignancy/infection	Sustained low donor reactivity and stable graft under reduced immunosuppression	Late clonal rebound, chronic injury, newly detected or clinically meaningful rebound DSA, recurrent severe infection, or inadequate vaccine recovery	Reverse minimization, treat cause, and continue lifetime surveillance

BCR, B-cell receptor; CMV, cytomegalovirus; dd-cfDNA, donor-derived cell-free DNA; dnDSA, *de novo* donor-specific antibody; DSA, donor-specific antibody; EBV, Epstein–Barr virus; eGFR, estimated glomerular filtration rate; ELISPOT, enzyme-linked immunospot; NK, natural killer; TCR, T-cell receptor; TREC, T-cell receptor excision circle.

A staged trial could use sequential gates. A conditioning cohort would first establish safety, engraftment, and target-compartment depletion without any planned immunosuppression reduction. A mechanistic cohort would then require prespecified evidence of donor-reactive reduction and naive-repertoire recovery while standard graft protection is maintained. Only participants meeting all cellular, humoral, injury, infection, and adherence gates would enter a randomized or within-patient minimization phase. Any isolated favorable biomarker would be insufficient. Because no validated composite exists for ISR, thresholds should be prespecified, analyzed continuously, and reviewed by an independent committee; biopsy-confirmed rejection, newly detected DSA or clinically meaningful rebound of tracked baseline DSA accompanied by graft injury, uncontrolled viral disease, or sustained loss of graft function should mandate reversal regardless of other signals.

## 9 Kidney-specific safety and ethical safeguards

Kidney candidates often have cardiovascular disease, vascular calcification, anemia, malnutrition, dialysis-access infection risk, and impaired vaccine responses. Conditioning can add mucositis, bacterial and fungal infection, viral reactivation, infertility, secondary malignancy, hemorrhagic cystitis, and organ toxicity. Perioperative kidney transplantation during cytopenia or severe lymphopenia may increase wound, thrombotic, infectious, and graft-injury risk. Trial sequencing must therefore avoid overlapping the nadir with major surgery unless supported by prior dose-finding and independent safety review.

Loss of pathogen-specific memory requires pre-reset immunization when feasible, post-AHSCT revaccination, quantitative immunoglobulin monitoring, and protocolized antimicrobial and viral prophylaxis (5, 8, 11). CMV, EBV, and BK surveillance should be more frequent than standard early kidney-transplant practice during delayed immune recovery. Live vaccines remain inappropriate during significant immunosuppression. Failure to recover protective vaccine responses or repeated opportunistic infection should stop immunosuppression minimization even when alloimmune biomarkers appear favorable.

Kidney transplantation generally confers a survival advantage over remaining on dialysis for suitable candidates, but the availability of dialysis does not neutralize its morbidity or make avoidable graft loss ethically acceptable (64). Early trials require independent data monitoring, explicit stopping boundaries, rapid access to biopsy and rescue immunosuppression, fertility counseling, long-term malignancy surveillance, and transparent consent that current evidence does not establish reliable drug-free tolerance.

Safety reporting should extend beyond the conventional 100-day AHSCT window. Relevant outcomes include hospitalization and intensive-care use, grade 3–4 infection, CMV/EBV/BK events, vaccine-serology recovery, hypogammaglobulinemia and replacement therapy, gonadal toxicity, cardiovascular events, malignancy, rejection phenotype, treatment-responsive vs. irreversible graft injury, and patient-reported burden. A regimen that reduces maintenance drug exposure but produces recurrent severe infection, prolonged functional immunodeficiency, or excess hospitalization would not represent clinically useful immune acceptance. Net benefit should be compared with the expected risk of remaining on dialysis or continuing standard immunosuppression for the same candidate.

## 10 Discussion

Autologous ISR offers a conceptually coherent alternative to donor-chimerism tolerance: rather than establish donor hematopoiesis, it attempts to reduce pre-existing recipient immune memory and guide recipient-derived reconstitution in the presence of a kidney allograft. Human autoimmune-disease data establish that repertoire renewal and regulatory rebalancing can occur. Preclinical studies using host-type, syngeneic, autologous, or congenic hematopoietic rescue show that alloimmune outcomes can be altered without intended donor chimerism; these terms describe different cell-source relationships and are not used interchangeably. RESTARRT, the liver-transplant primary sclerosing cholangitis pilot, and RESET demonstrate translational interest but provide limited, indirect, or not-yet-reported efficacy evidence for kidney-allograft tolerance.

The revised model therefore makes no claim that AHSCT routinely induces tolerance. Its value is as a falsifiable experimental framework. It succeeds only if a regimen produces donor-specific changes that are separable from global immunodeficiency and that permit safer immunosuppression reduction without humoral, cellular, innate, molecular, or histologic graft injury. Failure of thymic output, dominance of residual memory, persistent plasma-cell antibody, NK missing-self injury, or loss of pathogen immunity are predicted failure modes, not peripheral caveats.

The most informative next step is not a large withdrawal trial. It is a staged mechanistic program: first, define compartment-specific pharmacodynamics and safety in an appropriate population; second, verify naive repertoire renewal and donor-specific hyporesponsiveness under full graft protection; third, test conservative biomarker-gated minimization; and only then consider withdrawal in exceptional responders. Comparison against allocation, paired donation, desensitization, plasma-cell-directed therapy, and costimulation-blockade minimization is necessary to demonstrate clinical value.

## 11 Conclusion

AHSCT-based immune system reset is a plausible but unproven route to kidney-allograft acceptance. The central scientific question is whether recipient-derived immune reconstruction can produce measurable donor-specific hyporesponsiveness while preserving pathogen immunity and graft integrity. Current evidence supports carefully selected, biomarker-intensive mechanistic trials with explicit proceed, hold, and rescue rules. It does not support routine AHSCT for kidney-transplant tolerance or unmonitored immunosuppression withdrawal.
